# Identification of Failure Origin Through Testing and the Weibull Risk-of-Rupture

**DOI:** 10.6028/jres.099.048

**Published:** 1994

**Authors:** John A. Tesk, Martin Y. M. Chiang, Spurgeon M. Keeny, Jun Tang, Yuuji Sato

**Affiliations:** National Institute of Standards and Technology, Gaithersburg, MD 20899; Shanghai Pharmaceutical Industry Design Institute, Shanghai, China; Hiroshima University School of Dentistry, Hiroshima, Japan

**Keywords:** failure analysis, failure in bending, failure origin, failure stress, failure stress and size effect, finite element analysts, finite clement stress, origin of failure, Weibull analysis, Weibull hazard function, Weibull risk-of-rupture function

## Abstract

The stress distribution in bond layers of two different thicknesses (50 μm and 200 μm) was calculated by finite element analysis for pairs of rectangular cross section metal bars bonded to each other and subjected to four point bending. These stresses were used to aid in identification of the failure origin by use of the Weibull risk-of-rupture (RR) function. By use of the stress distributions, the characteristic strength from 50 μm bond test specimens could be correlated with that for 200 μm bond test specimens when the failure was assumed to have an interfacial origin. The finite element meshes were refined twice and the ratios of characteristic strengths were recalculated and remained virtually unchanged by each of the mesh refinements. Hence, the identification of the interface as the failure origin remained consistent. Further, the use of stresses extrapolated to zero mesh size also produced the same ratios. Therefore, the RR calculations do not appear to be sensitive to the mesh sizes used for the stress calculations when the meshes are comparable or when changed in a comparable manner. The results show this method can be consistent and a useful adjunct for identification of failure origins.

## 1. Introduction

In previous work [1,2] bending tests were conducted on adhesively bonded specimens of a dental alloy. The purpose was to determine:

- how much the bond thickness influenced the test results;
- whether the failure origins appeared to be the same for the two different bond thicknesses employed;
- failure origin through analysis using the risk-of-rupture (RR) function.

In this paper, the finite element method was used to arrive at the stress distributions used for the RR analyses that employ the Weibull risk-of-rupture function^1^ [3]. An ancillary purpose, therefore, was to ascertain how sensitive the analyses were to the fineness of the finite-element mesh and, hence, whether the method can be applied with confidence to the analyses conducted for a, b, and c sbove.

## 2. Materials and Methods

The failure of brittle materials is typically catastrophic and in many instances the failure stresses obtained from a set of test specimens follow a Weibull distribution. For a homogeneous isotropic material subjected to a uniform tensile stress, *σ*, the probability of failure, *P(σ)*, is given by:

$$P\left(\sigma \right)=1-\exp-\left[\delta {\left(\sigma /\sigma_{0,u}\right)}^{m}\right],$$ (1)

where *σ*_0_,_u_ is a characteristic strength for a specimen of unit dimension; *m* is a shape factor (Weibull modulus); and *δ* is a size factor (the ratio of the failure originating dimension to a unit dimension of the same kind) and represents the volume, *V*, area, *A*, or other dimension in which reside the flaws from which the failure originates [3] Eq. (1) is often written as:

$$P\left(\sigma \right)=1-\exp-[{\left(\sigma /\sigma_{0}\right)}^{m}],$$ (1a)

where the size of the specimen, *δ*, is subsumed into *σ*_0_. This form of the equation is commonly used when analyzing test data and the effects of specimen size are ignored. It is clear from Eq. (1) that for specimens of two sizes, *σ*_1_ and *σ*_2,_ with the same failure origins and presenting the same distribution of failure stresses (*m*_1_=*m*_2_), there will be two different values of *σ*_0_ for Eq. (1a), with the larger size, call it *δ*_1_, leading to a value, *σ*_0.1_, that is less than*σ*_0.2_

For such specimen sets, the relation between the characteristic strengths calculated by Eq. (1a) is [4]:

$$\sigma_{0.2}=\sigma_{0.1}\;{\left[\delta_{1}/\delta_{2}\right]}^{1/m}.$$ (2)

For a nonuniform tensile stress field, a more general form of Eq. (1) is necessary:

$$P(\sigma )=1-\exp-\left[\int_{\delta }{\left(\sigma /\sigma_{\upsilon }\right)}^{m}d\delta \right],$$ (3)

where the region of integration over *δ* is the region critical to failure (rupture) and it can be in one, two, or three dimensions. Then the relationship between the values of *σ*_0_) [Eq. (1a)] as determined from experiments on sets of specimens having either one or the other of the bond thicknesses, is given by the ratios of the exponents of Eq. (3), i.e.,

$$\frac{{\left(\sigma /\sigma_{0.1}\right)}^{m}}{{\left(\sigma /\sigma_{0.2}\right)}^{m}}=\frac{\int_{\delta 1}{\left(\sigma_{1}/\sigma_{0.0}\right)}^{m}d\delta_{1}}{\int_{\delta 2}{\left(\sigma_{2}/\sigma_{0.0}\right)}^{m}d\delta_{2}}.$$ (4)

Canceling terms in *σ*_0.u_ on the right side and *σ* on the left side leads to

$$\frac{\sigma_{0.2}}{\sigma_{0.1}}={\left[\frac{\int_{\delta 1}{\left(\sigma_{1}\right)}^{m}d\delta_{1}}{\int_{\delta 2}{\left(\sigma_{2}\right)}^{m}d\delta_{2}}\right]}^{1/m}$$ (4a)

When *σ_i_*(*δ*) is not known as an explicit function, the relationship between *σ*_0_._1_ and *σ*_0_._2_ may, in principle, be approximated by [3]):

$$\sigma_{0.2}=\sigma_{0.1}\;{\left[\left(\sum_{j}\sigma_{1,j}^{m}\Delta \delta_{1,j}\right)/\left(\sum_{j}\sigma_{2,j}^{m}\Delta \delta_{2,j}\right)\right]}^{1/m},$$ (5)

and the validity of the approximation must be checked by computation. Here the summations are over all the elements considered to be involved with the failure (interface, volume, etc.) and the stresses can be evaluated by the finite-element method of analysis.

Note: The stress field in the bond region is typically three dimensional; the analyses of this paper utilize unidirectional tensile stresses because alterations to the principal stresses were found to be minor and may be ignored. We also note that for a variety of reasons (plasticity, change in composition, properties, or flaw populations) this analysis method may not apply for very thin bond layers approaching micrometers or less.

Each assumed failure origin for a specimen has its own specific *δ* with its associated stresses. When the ratios of volumes, surface areas, interface areas, edge lengths etc. (any dimensions containing the flaws from which failures may originate) are properly chosen to be different for experimental tests, only one set of *δ_i_*’s, *σ_i_*’*s*, *δ_j_*’*s*, and *σ_j_*’s should produce coincidence between the experimentally determined ratio of *σ*_0_’s and the ratios of either the integrals shown by Eq, (4a) or the summations as shown in Eq. (5).

As with any analysis employing the finite element (FE) method for determination of the stresses, a critical question arises as to the FE-mesh sensitivity of Eq. (4). If sensitive, then the method would not, in actuality, be useful for the correlation of results from differently sized specimens.

**Fig. 1:**
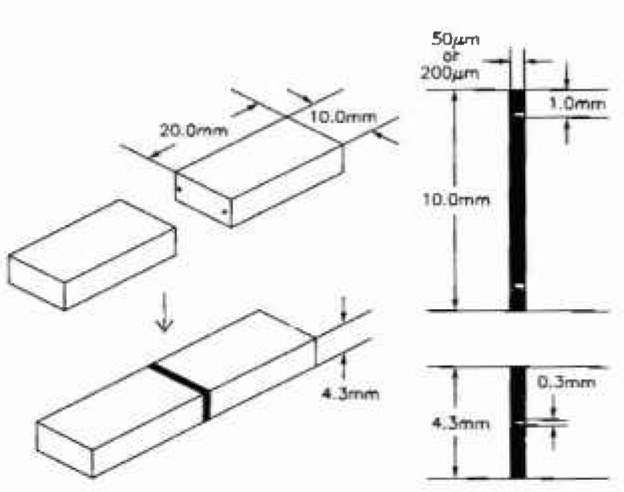
Specimen used for bond testing. The shaded area represents the bonding material between rectangular bearne of alloy that were bonded together. Two small projections were used to control the width of the bond at either 50 μm or 200 μm.

To provide insight into the ability of this approach to identify sources of failure, rectangular bond specimens as shown in Fig. 1 were prepared for testing in four-point bending, with either 50 μm or 200 μm bond-thicknesses, Rectangular bars of the bulk bonding material were also tested in three-point bending. The details of specimen preparation were given in a presentation by Keeny et al. [1]. The number of specimens and the results for each test series are shown in Table 1.

**Fig. 2:**
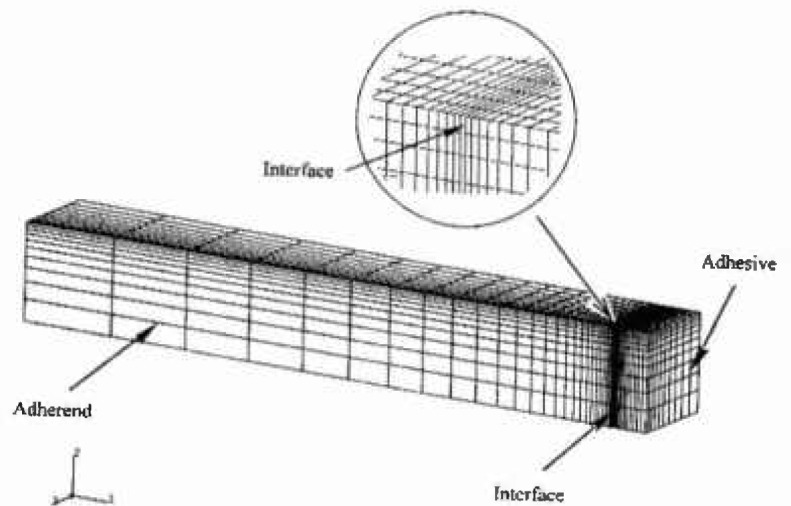
A 1/8 section of a three-dimensional model for finite element calculations of stress. The specimens (Fig. 1) had three planes of symmetry.

**Fig. 3:**
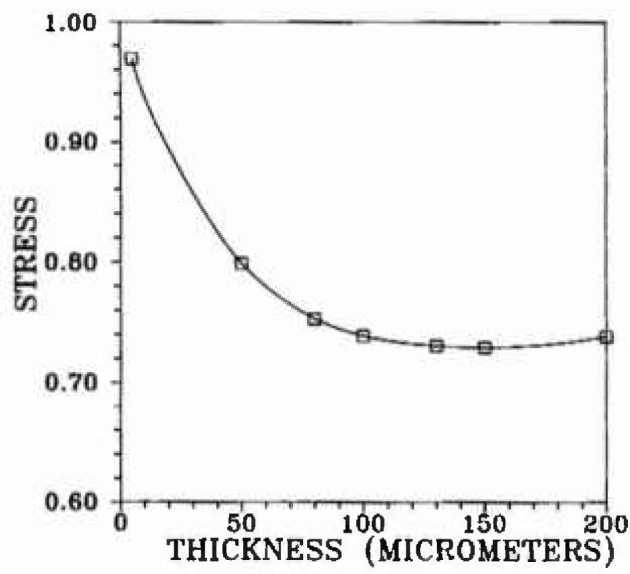
Result of finite element calculation of the near-surface bending stress at the bond midplane that bisects the bend specimens into symmetrical halves.

**Fig. 4:**
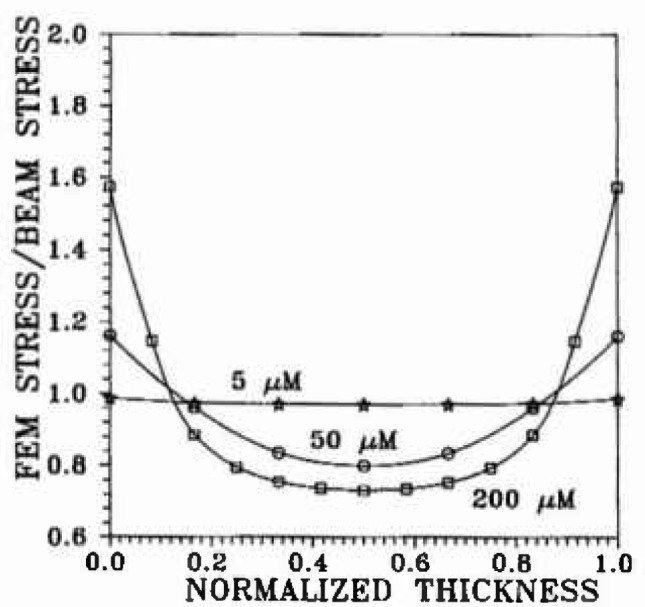
Surface bending stress across the surface of the bond material, from one interface junction to the opposite one, as calculated fur three thicknesses, The 5 μm thickness is presented to illustrate the trend toward beam stress calculations as the thickness approaches zero. The deviations from beam theory calculations arc appreciable for thick bond specimens, showing the need to use the more robust finite clement method for the failure analysis employing the RR function.

**Fig. 5:**
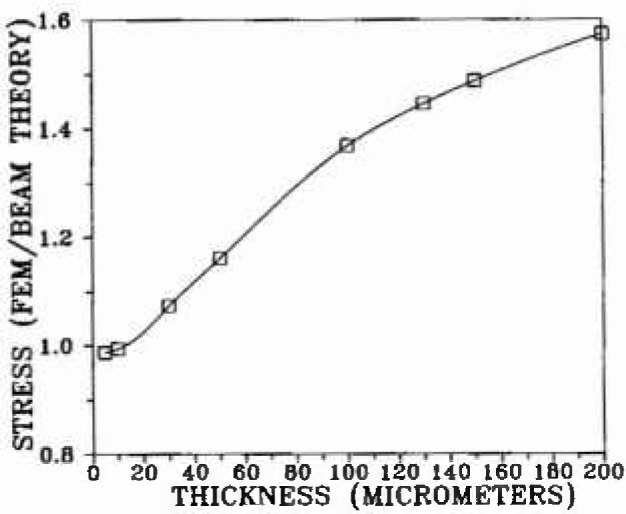
Finite element calculated stress for the adherend-adherent interface surface junction line, illustrating the dramatic effect of bond thickness on interface stresses.

A three-dimensional, finite-element elasticity model^2^ was used for evaluation of the stress distribution throughout the volume of the bond region. The original bond model (Fig. 2) consisted of 2,197 elements in one quadrant of the specimen which had three planes of symmetry. Subsequent refinements of interface and surface elements led to elements 1/2 and 1/3 the original size. The validity of the model was checked by comparison of the finite-element results for a homogeneous beam with the analytical solution. Examples of how the bending tensile stresses change as a function of the thickness of the bond layer are shown in Figs. 3, 4, and 5, for which *E*_b_ ≡ *E_a_*/50 where: *E*_b_ is Young’s modulus for the bonding material; and *E_a_* is Young’s modulus for the alloy.

If the failure stresses are referenced to the stresses along the surface, the ratio of the operative (effective) dimensions, *δ*, from which the failures originate are given by Eq. (6):

$$\frac{\delta_{eff,1}}{\delta_{eff,2}}={\left(\frac{\sigma_{\text{R},2}}{\sigma_{\text{R},1}}\right)}^{m}\frac{\sum_{j}\sigma_{1,j}^{m}\Delta \delta_{1,j}}{\sum_{j}\sigma_{2,j}^{m}\Delta \delta_{2,j}}$$ (6)

where *σ*_R_ denotes the reference stress.

For these calculations the bending stress at the surface was used as the reference stress for calculations of bending strength and *σ*_R,2_=*σ*_R,1_. Then, from Eq. (2) and Eq. (6)

$$\sigma_{0.2}/\sigma_{0.1}={\left(\delta_{\mathrm{eff},1}/\delta_{\mathrm{eff},2}\right)}^{1/m}={\left[\frac{\sum_{j}\sigma_{1,j}^{m}\Delta \delta_{1,j}}{\sum_{j}\sigma_{2,j}^{m}\Delta \delta_{2,j}}\right]}^{1/m},$$ (7)

which is equivalent to Eq. (5).

## 3. Results

By the use of the right-hand side of Eq. (7) and the finite-element-derived stress distributions, characteristic strength ratios were calculated for four potential regions (Table 2) where the failure of the bond could originate, i.e., volume, surface, interface, and interface-line-junction failure origins. These were then compared with the results obtained from the left hand side of Eq. (7). For these calculations, a value of *m* = 11.5 was used [in Table 1, *m* was obtained from Newton-Raphson iteration for fitting experimental data to Eq. (1)].

The ratio of the experimental characteristic strengths has a 95% confidence range of 0.955 to 1.11. When this ratio is compared with ratios calculated from the finite element analyses, the interface (Table 2) is identified as the origin of failures, with all other failure origins excluded. The row with the next closest match of strength ratios is that for volume failures which, with a ratio of 1.12, lies just outside the 95% confidence range, so this argument, by itself, is somewhat unconvincing. However, volume failures are ruled out because the *m* value of 11.5 from the bond tests differs, at the 90% confidence level, from the value of *m* =6.8 which was obtained from the bulk specimen test data. The bulk specimens can fail only by volume or surface failures. The strength ratio calculations for bond specimens rule out surface failures. The *m* value differences then are used to rule out volume failures.

Hence, the most reasonable explanation is that the bond specimens fail by interfacial failures. This is consistent with features of the failed specimens which always presented regions showing interfacial debonding.

There is some possibility that the strength of the bond itself would depend on the bond thickness due to a change in material response (i.e., formation of plasticity). Such effects obviously cannot be dealt with by the linear elastic analysis presented and within the confines of this analysis, interfacially initiated failure is concluded.

## 4. Summary

An analysis by Weibull RR for bonded specimens of two different sizes tested in bending has shown:

1. Correlations between characteristic strengths, *σ*_0_’s, were possible through the use of finite-element derived stresses in the RR analysis.
2. The correlations were not sensitive to the particular mesh chosen.
3. For the interface, surface, and interface-surface junction line, the stress ratio calculations employing the element centroid stresses are not significantly changed by use of stresses from extrapolations to the interface. The largest difference is for the interface-surface junction line and these are shown in Table 2.
4. Because the absolute magnitude of each RR calculation changes, mesh of the same size and configuration must be used for each set of comparisons.
5. The origins of failures can be determined by suitable testing and analysis of different size bond specimens and bulk specimens of the bonding material. This involves the use of a combined approach, analysis of the *σ*_0_’s and *m* values.
6. The determination of failure origin by this approach can be useful for focusing attention on the proper parameters if improvements in system strength or performance are sought.

## Figures and Tables

**Table 1:** Uncorrected Weibull parameters and mean strengths

Test	Gap (μm)	*N* a	*σ*_0_ (MPa) {range 95%}^b^	*m* {range 95%}
4-Pt Bend	50	25	110 {106–115}	11.3 {8.2–14.0}
	200	25	107 {105–111}	11.5 {8.3–14.2}
3-Pt Bend	Bars of bulk bond material	54	85 {81–89}	6.8 {5.5–7.93}

a *N* = the number of specimens.

b {} = the associated confidence bounds on *σ*_0_ and *m* as determined from the data.

**Table 2:** Ratios of characteristic strengths calculated from risks of rupture: (strength, 200 μm)/(strength, 50 μm). Four-Point-bending

Assumed failure origin	Coarse mesh	Refined mesh 1	Refined mesh 2
Volume	1.12	1.12	1.12
Interface	.995	1.01	1.06
Surface	1.26	1.36	1.32
Interface-surface junction line	1.24	1.25	1.41
Interface-surface junction line (extrapolated)^a^	1.38	1.37	1.37

a Obtained from extrapolations of finite element derived stresses at ccntroids to the interface between alloy and bond layer.

## Footnotes

1 Today, a risk-of-rupture function, defined by Weibull, would be recognized as a hazard function.

2 Developed at NIST.
